# When viruses unite forces: introducing a novel system to study tomato chlorosis virus

**DOI:** 10.1093/plphys/kiag228

**Published:** 2026-04-21

**Authors:** Mireia Uranga

**Affiliations:** Assistant Features Editor, Plant Physiology, American Society of Plant Biologists, Rockville, MD 20855-2768, United States; Institute for Integrative Systems Biology (I2SysBio), Universitat de València-CSIC, Paterna, Valencia 46908, Spain

People love tomato. Widely used in sauces, stews, and soups and as a fresh topping in salads, it is a cornerstone ingredient in Mediterranean and other Western cuisines. Tomato is ranked as the most produced vegetable crop worldwide (FAO, 2024), but tomato production is currently threatened by emerging viral diseases. The first report of tomato chlorosis virus (ToCV; genus *Crinivirus*) dates back to the mid-1990s, when greenhouse-grown tomato plants from Florida, USA, exhibited a “yellow leaf disorder” (Wisler et al. 1998). Characteristic symptoms included the development of chlorotic areas in lower leaves that gradually progressed to interveinal yellowing in the upper part of the plant. In advanced stages, reddish-brown necrotic spots appeared in leaves, together with anthocyanin accumulation on the abaxial side. The loss of photosynthetic area affected plant vigor and fruit development, eventually leading to significant yield reduction. ToCV is naturally transmitted by whitefly vectors belonging to the genera *Bemisia* and *Trialeurodes* and is now present in more than 35 countries (Fiallo-Olivé and Navas-Castillo 2019). In the absence of resistant tomato cultivars, chemical control of the insect vectors is ineffective in avoiding disease expansion to new geographical regions. Thus, a deep understanding of ToCV pathogenesis is urgently needed to develop effective control strategies.

Researchers require a simple and effective method to reliably recreate ToCV infection in experimental conditions. Whitefly-mediated transmission is regarded as the standard procedure to perform ToCV inoculations in confined environments, albeit time-consuming and labor intensive (Orfanidou et al. 2016). Alternative procedures include graft-inoculation with virus-infected plants (Çevik et al. 2019) and *Agrobacterium*-mediated delivery of binary plasmids harboring ToCV infectious clones (Orílio et al. 2014; Kwon et al. 2024). The latter approach has been proven to trigger viral disease in *Nicotiana benthamiana* but not in tomato, probably as a result of inefficient delivery and/or low plasmid replication that prevents sufficient ToCV accumulation.

Recently in *Plant Physiology*, Wang et al. (2026) developed a less virulent tobacco rattle virus (TRV; genus *Tobravirus*) vector to effectively induce ToCV infection in tomato (Fig. 1a). TRV is commonly used in virus-induced gene silencing for performing gene function studies in plants, thanks to a wide host range of more than 50 plant families (including Solanaceae) and its capacity to cause mild infection symptoms by reaching all plant tissues (Shi et al. 2021). In their work, the authors reverse-amplified ToCV RNA1 and RNA2 from symptomatic greenhouse-grown tomato material and then integrated the corresponding full-length cDNA fragments into TRV (henceforth named pTRV2-GW-RNA1 and pTRV2GW-RNA2).

**Figure 1:**
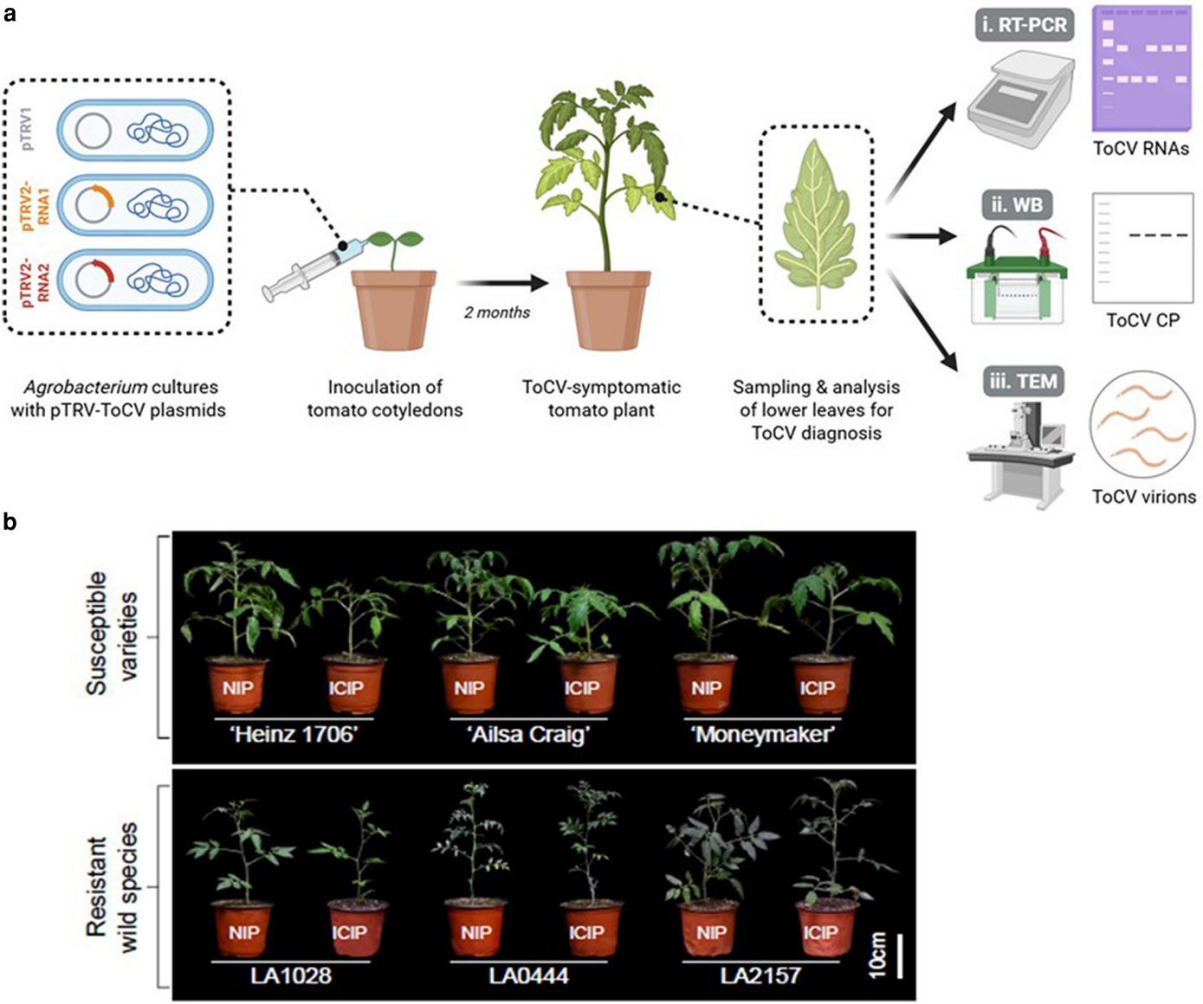
ToCV infectious clones produced by a TRV vector enable viral infection in tomato. a) *Agrobacterium* suspensions carrying pTRV1, pTRV2GW-RNA1, and pTRV2GW-RNA2 are mixed in equal proportions and infiltrated into tomato cotyledons. The inoculated tomato plants are placed in a growth chamber and monitored for the development of viral infection. Two months later, the lower leaves are sampled, and the presence of ToCV is analyzed by (i) RT-PCR amplification of viral RNA1 and RNA2; (ii) western blot (WB) detection of the viral capsid protein (CP); and (iii) the characterization of viral particles by transmission electron microscopy (TEM). b) Inoculation with ToCV infectious clones is a practical method for identifying ToCV resistance among tomato genotypes. NIP, non-inoculated plant; ICIP, infectious clone inoculated plant. Adapted from Figure 5, Wang et al. (2026), and created in BioRender.

*Agrobacterium* suspensions carrying pTRV1, pTRV2GW-RNA1, and pTRV2GW-RNA2 were mixed in equal proportions to form ToCV infectious clones and infiltrated into 10-d-old tomato cotyledons. At 21 d post-inoculation, RT-PCR analysis revealed that regions of ToCV RNA1 and RNA2 were present in the lower leaves of the inoculated plants. A successful infection was later confirmed by the detection of the capsid protein via western blot, and ToCV aggregates were also observed using transmission microscopy. Two months after the inoculation, infected tomato plants exhibited stunted growth, chlorosis, and anthocyanin accumulation in lower leaves together with a reduction of the leaf photosynthetic and antioxidant capacities. When plants reached the reproductive stage, fruits were significantly smaller in size than those of non-inoculated plants and could not fully turn red, reflecting impaired fruit development and ripening. Similar to what is observed upon graft-inoculation, these findings demonstrate that the TRV-derived ToCV infectious clones can effectively trigger viral disease in tomato.

Although several wild tomato species have been reported to display mild symptoms upon ToCV infection, so far there are no commercially available ToCV-resistant or -tolerant tomato cultivars (Gao et al. 2025). Thus, farmers would greatly benefit from a method that enables a rapid identification of ToCV-resistant plant material. For this aim, Wang et al. (2026) inoculated the ToCV infectious clones into susceptible tomato cultivars (Heinz 1706, Ailsa Craig, and Moneymaker) and resistant genotypes from wild tomato species (*Solanum chmielewskii* LA1028, *S. corneliomulleri* LA0444, and *S. arcanum* LA2157) (Fig. 1b). As expected, commercial cultivars were considerably shorter than the non-inoculated controls at 2 mo after the inoculation. Additionally, lower leaves developed typical ToCV infection symptoms and showed lower photosynthetic and antioxidant activities. Conversely, the wild tomato genotypes lacked noticeable phenotypical changes and accumulated lower levels of the viral capsid protein than commercial varieties, as confirmed via western blot. Therefore, the ToCV infectious clones constitute a suitable tool for screening virus resistance among tomato germplasms.

Since its discovery in the mid-1990s, ToCV has expanded worldwide due to inappropriate agricultural practices, the international trade of plant materials, and the establishment of its insect vectors in temperate climates. Moreover, the occurrence of mixed viral infections in the field (mainly between ToCV and tomato yellow leaf curl virus, TYLCV) enhances disease severity and adds complexity to the epidemiology (Li et al. 2021). The recombinant TRV vector developed by Wang et al., (2026) generates ToCV infectious clones that can successfully infect tomato, providing an ideal system to improve our understanding of ToCV pathogenesis and accelerate resistant breeding. Addressing all these questions will not only contribute to improving control strategies against ToCV but will also open new venues for creating highly infectious viral clones for other pathosystems.

## Data Availability

None required.
